# Genome-wide identification of Diacylglycerol Acyltransferases (DGAT) family genes influencing Milk production in Buffalo

**DOI:** 10.1186/s12863-020-0832-y

**Published:** 2020-03-06

**Authors:** Jiajia Liu, Zhiquan Wang, Jun Li, Hui Li, Liguo Yang

**Affiliations:** 1 Key Lab of Agricultural Animal Genetics, Breeding and Reproduction of Ministry of Education, Huazhong Agriculture University, Wuhan, China; 2 School of Biological Science and Technology, University of Jinan, Jinan, China; 3 Department of Agricultural, Food, and Nutritional Sciences, University of Alberta, Edmonton, Canada; 4 Department of Immunology, Zunyi Medical College, Zunyi, China

**Keywords:** DGAT family, *DGAT1*, *DGAT2*, Milk production traits, Buffalo genome

## Abstract

**Background:**

The diacylglycerol acyltransferases (DGAT) are a vital group of enzymes in catalyzing triacylglycerol biosynthesis. DGAT genes like *DGAT1* and *DGAT2*, have been identified as two functional candidate genes affecting milk production traits, especially for fat content in milk. Buffalo milk is famous for its excellent quality, which is rich in fat and protein content. Therefore, this study aimed to characterize DGAT family genes in buffalo and to find candidate markers or DGAT genes influencing lactation performance.

**Results:**

We performed a genome-wide study and identified eight DGAT genes in buffalo. All the DGAT genes classified into two distinct clades (DGAT1 and DGAT2 subfamily) based on their phylogenetic relationships and structural features. Chromosome localization displayed eight buffalo DGAT genes distributed on five chromosomes. Collinearity analysis revealed that the DGAT family genes were extensive homologous between buffalo and cattle. Afterward, we discovered genetic variants loci within the genomic regions that DGAT genes located in buffalo. Seven haplotype blocks were constructed and were associated with buffalo milk production traits. Single marker association analyses revealed four most significant single nucleotide polymorphisms (SNPs) mainly affecting milk protein percentage or milk fat yield in buffalo. Genes functional analysis indicated that these DGAT family genes could influence lactation performance in the mammal through regulating lipid metabolism.

**Conclusion:**

In the present study, we performed a comprehensive analysis for the DGAT family genes in buffalo, which including identification, structural characterization, phylogenetic classification, chromosomal distribution, collinearity analysis, association analysis, and functional analysis. These findings provide useful information for an in-depth study to determine the role of DGAT family gens play in the regulation of milk production and milk quality improvement in buffalo.

## Background

Water buffalo is the second most extensive resource of milk supply around the world, and it is well known for its high milk quality with higher fat contents compared with cattle milk (6.4–8.0% vs. 4.1–5.0%) [1]. They also convert the low-quality indigenous grasses into milk more efficiently than dairy cows with lower methane emissions. However, buffalo milk yield was known to be much lower than that of cow [2], which largely restricts the development of the dairy buffalo industry. The milk traits are complex quantitative traits with moderate heritability [3–5]. With the application of genome-wide association studies for mapping genes for complex traits in domestic animals, several necessary genes related to lactation phenotypes have been identified. For example, the *DGAT1* gene is one of the crucial candidate genes and for milk traits in animals [6, 7]. The missense mutation K232A in *DGAT1* showed to have significant effects on milk traits in cattle, which the A allele was associated with increased milk yield, whereas the K allele with increased fat and protein concentrations [8, 9]. Moreover, the *DGAT1* K232A was also contributed to influence the fatty acids contents of milk in both cattle and buffalo [10]. Polymorphisms of the *DGAT1* were found to be fixed on allele K at K232A locus in some buffalo breeds, which were considered to be the primary responsibility for the high milk fat in buffalo [4, 11, 12]. However, it’s hard to credit most of the effects to one single gene *DGAT1*, since the milk production traits are polygenic traits, a series of genes may be involved in the process, especially in genes belonging to the same family, which have similar sequence and likely common evolutionary origin and similar function.

The diacylglycerol acyltransferases (DGAT) enzymes are essential ones that control the final rate-limiting step of triacylglycerol biosynthesis for the significant milk lipid. Presently, four distinct DGAT functional subfamilies, including DGAT1, DGAT2, DGAT3, and WAX-DGAT, have been discovered in different organisms [13–15]. Among them, only DGAT1 and DGAT2 enzymes have been detected in animals, which was shown to play non-redundant roles in triacylglycerides synthesis [16]. Of these, the *DGAT1* gene was the first recognized gene encoding a protein with DGAT enzyme activity. The DGAT1-knockout mice were alive and could still synthesize triglycerides [17]. This study well validated that DGAT-like activity found in the enzymes encoded by other genes leading to the discovery of DGAT2 enzyme. *DGAT2* gene is closely related to the candidate for quantitative traits, and it was associated with lipid synthesis and storage in eukaryotes. The analysis of genetic variation at the *DGAT2* gene can be used to evaluate milk productive traits in buffalo [18]. Relevant investigation in goat showed that *DGAT2* was an active candidate gene affecting goat milk yield and fat percentage [19]. Novel associations detected between *DGAT2* genetic variability and the milk yield in cattle [20]. Beside of *DGAT2*, this DGAT2 subgroup contains other members, including *MOGAT1, MOGAT2, MOGAT3, AWAT1,* and *AWAT2* [16, 21]. The members of the DGAT2 subfamily are high priority candidate genes for quantitative traits related to dietary fat uptake and triglyceride synthesis and storage in animals [21]. These findings inspired our curiosity to understand the effect of DGAT family genes on milk traits in buffalo.

The recent completion of the buffalo genome sequence made the genome-wide identification of buffalo family genes possible [22]. Therefore, the present study aimed to detect DGAT family genes in the buffalo genome, and then perform a detailed analysis of the classification, physicochemical properties, phylogenetic analysis, structural features, and functional analysis. Furthermore, association analysis of DGAT family genes with buffalo milk production traits was performed in order to identify essential markers or genomic regions affecting buffalo milk. Our study provided a deep insight into DGAT family genes that influence milk production traits, which is essential for future improvement of milk quality and quantity in the buffalo breeding industry.

## Results

### Identification of the members in the DGAT family

To identify the DGAT family members, we used 21 verified DGAT amino acid sequences from bovine (*Bos taurus*, 3), human (*Homo sapiens*, 9), mouse (*Mus musculus*, 7) and rat (*Rattus norvegicus*, 2) as the query for genome-wide detection of the homologous sequences (Additional file 1). As a result, 24 non-redundant protein sequences encoded by eight *DGAT* genes (DGATs), including *DGAT1, DGAT2, DGAT2L1/MOGAT1, DGAT2L3/AWAT1, DGAT2L4/AWAT2, DGAT2L5/MOGAT2, DGAT2L7/MOGAT3*, and *DGAT2L6* were identified in *Bubalus bubalis* (Table 1). In parallel, 15 DGAT protein homologous sequences of these eight DGAT genes, were recognized in *Bos taurus* (Additional file 2).

**Table 1:** Details of Genome-wide identified DGAT family members in *Bubalus bubalis*

Genes (Transcripts)	Protein isoform	Protein identifier	Amino acids	Mw/kDa	pI	Production
DGAT1 (8)	DGAT1	NP_001277831.1	489	55.46	9.61	Diacylglycerol O-acyltransferase 1
	DGAT1.1	XP_025120523.1	519	58.24	9.35	Diacylglycerol O-acyltransferase 1 isoform X1
	DGAT1.2	XP_025120524.1	504	56.44	9.37	Diacylglycerol O-acyltransferase 1 isoform X2
	DGAT1.3	XP_025120525.1	499	57.44	9.53	Diacylglycerol O-acyltransferase 1 isoform X3
	DGAT1.4	XP_025120526.1	497	55.89	9.22	Diacylglycerol O-acyltransferase 1 isoform X4
	DGAT1.5	XP_025120527.1	474	49.72	8.32	Diacylglycerol O-acyltransferase 1 isoform X5
	DGAT1.6	XP_006064685.2	467	53.25	9.45	Diacylglycerol O-acyltransferase 1 isoform X6
	DGAT1.7	XP_025120528.1	459	48.28	8.32	Diacylglycerol O-acyltransferase 1 isoform X7
DGAT2 (2)	DGAT2	XP_006045249.1	361	40.95	9.41	Diacylglycerol O-acyltransferase 2 isoform X1
	DGAT2.1	XP_025122178.1	318	36.17	9.38	Diacylglycerol O-acyltransferase 2 isoform X2
DGAT2L1/ MOGAT1 (1)	DGAT2L1	XP_006079705.1	335	39.09	9.34	2-acylglycerol O-acyltransferase 1
DGAT2L3/ AWAT1 (1)	DGAT2L3	XP_006074252.2	328	37.85	9.26	Acyl-coa wax alcohol acyltransferase 1
DGAT2L4/ AWAT2 (2)	DGAT2L4	XP_006074246.1	282	32.14	9.41	Acyl-coa wax alcohol acyltransferase 2 isoform X1
	DGAT2L4.1	XP_006074245.1	333	37.93	9.41	Acyl-coa wax alcohol acyltransferase 2 isoform X2
DGAT2L5/ MOGAT2 (5)	DGAT2L5	XP_006074149.1	334	38.76	9.80	2-acylglycerol O-acyltransferase 2
	DGAT2L5.1	XP_025121484.1	333	38.47	8.70	2-acylglycerol O-acyltransferase 2-like
	DGAT2L5.2	XP_025121673.1	334	38.63	9.14	2-acylglycerol O-acyltransferase 2-like
	DGAT2L5.3	XP_006074155.1	328	37.89	9.48	2-acylglycerol O-acyltransferase 2-like
	DGAT2L5.4	XP_006045248.2	363	41.88	9.63	2-acylglycerol O-acyltransferase 2-like
DGAT2L6 (1)	DGAT2L6	XP_025132892.1	600	68.21	8.10	Diacylglycerol O-acyltransferase 2-like protein 6
DGAT2L7/ MOGAT3 (4)	DGAT2L7	XP_025130795.1	338	38.50	8.97	2-acylglycerol O-acyltransferase 3
	DGAT2L7.1	XP_025131217.1	333	37.50	9.26	2-acylglycerol O-acyltransferase 3-like isoform X1
	DGAT2L7.2	XP_025131218.1	329	37.48	8.84	2-acylglycerol O-acyltransferase 3-like isoform X2
	DGAT2L7.3	XP_006047367.2	365	41.40	8.83	2-acylglycerol O-acyltransferase 3-like

*Mw* molecular weight, *pI* isoelectric point

The length of amino acid sequences of 24 buffalo DGAT protein isoforms ranged from 282 (DGAT2L4) to 600 (DGAT2L6), and their molecular weight was 32.14–68.21 kDa that correlated well with the protein length. The value of isoelectric points was higher than 8.0, which indicated that buffalo DGAT proteins containing more basic amino acids than acidic amino acids. Moreover, we detected eight DGAT1 protein isoforms (DGAT1 – DGAT1.7) all contained the Membrane-Bound *O-acyl* Transferase (MBOAT) conserved domain (Additional file 3). The other seven DGATs produced amino acid sequences mainly harbored the Diacylglycerol Acyltransferase (DAGAT) or Lysophosphatidic Acyltransferase (LPLAT) conserved domain. Results of subcellular localization prediction showed buffalo DGAT proteins all located in the endoplasmic reticulum membrane. Since triacylglycerol biosynthesis occurs mainly at the endoplasmic reticulum, DGAT enzymes in the membrane are conducive to the synthesis of catalytic lipids [23].

### Structural features of buffalo DGAT family members

**Fig. 1:**
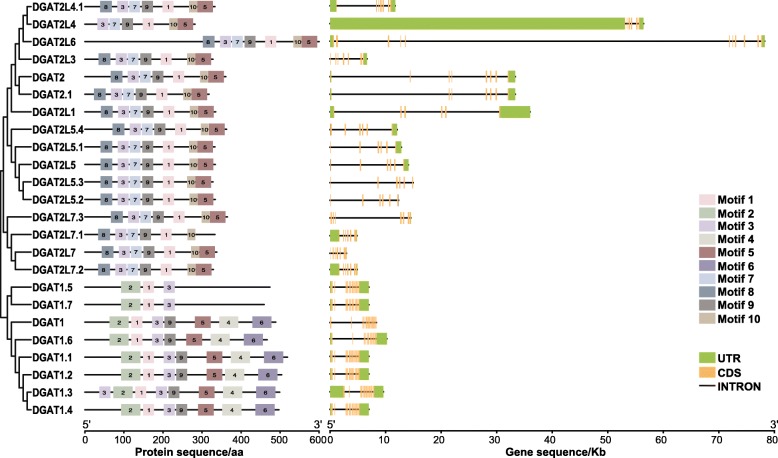
Characterizations of the identified DGAT proteins and genes isoforms in buffalo. The phylogenetic tree (left) was constructed by neighbor-Joining method. Structure of amino acid sequences (middle), boxes with different colors represent ten different conserved motifs. In the gene structure plot (right), green box, black line and orange box represent untranslated region (UTR), intron and coding sequencing (CDS), respectively

In order to explore the structural characteristics of buffalo DGAT proteins and genes, the conserved motifs and gene structures were projected based on their phylogenetic relationships (Fig. 1). As the results have shown, the buffalo DGAT family members initially categorized into two main clades: DGAT1 and DGAT2 subfamily. Among eight buffalo DGAT genes, *DGAT1* gene belongs to DGAT1 subfamily, and the other seven DGATs all assigned to DGAT2 subfamily. In DGAT1 subfamily, five conserved domains including Motif 2, 1, 3, 9, 5, 4, and 6 were shared among the majority protein isoforms of DGAT1, DGAT1.1, DGAT1.2, DGAT1.3, DGAT1.4 and DGAT1.6. The prediction of their gene structures was highly similar in the coding areas, which contained 17 coding sequences (CDSs) and 18 introns. While the length and layout of 3′ untranslated region (UTR) and 5′ UTR were various in the non-coding areas. On the other side, for DGAT2 subfamily members, there were seven conserved motifs (Motif 8, 3, 7, 9, 1, 10, and 5) in almost all of their amino acid sequences. Gene structural analysis discovered, although the introns and UTRs structure varied greatly, the import coding sequences were consistent among the nucleotide sequences of all DGAT2 subfamily genes.

The same conserved patterns among DGAT1 and DGAT2 subfamily proteins were Motif 1, Motif 3, Motif 5 and Motif 9, which composited of 29, 29, 41 and 29 amino acids, respectively (Additional file 4). Meanwhile, Motif 2, 4, and 6 were three unique conserved amino acid sequences of DGAT1 subfamily, and DGAT2 subfamily-specific conserved motifs included in Motif 7, 8, and 10. subsequently, to better discern the structural features of buffalo DGATs, we predicted the transmembrane helixes for eight DGAT enzymes (Additional file 5). The topological studies displayed that DGAT1 subfamily protein contained ten transmembrane domains, and DGAT2 subfamily proteins generally have 3–5 transmembrane structures. Besides, DGAT1 protein has N-terminus oriented towards the cytosol with the C-terminal region, which accounts for approximately 50% of the protein, and is present in the endoplasmic reticulum lumen.

### Phylogenetic relationship of DGAT proteins in different organisms

**Fig. 2:**
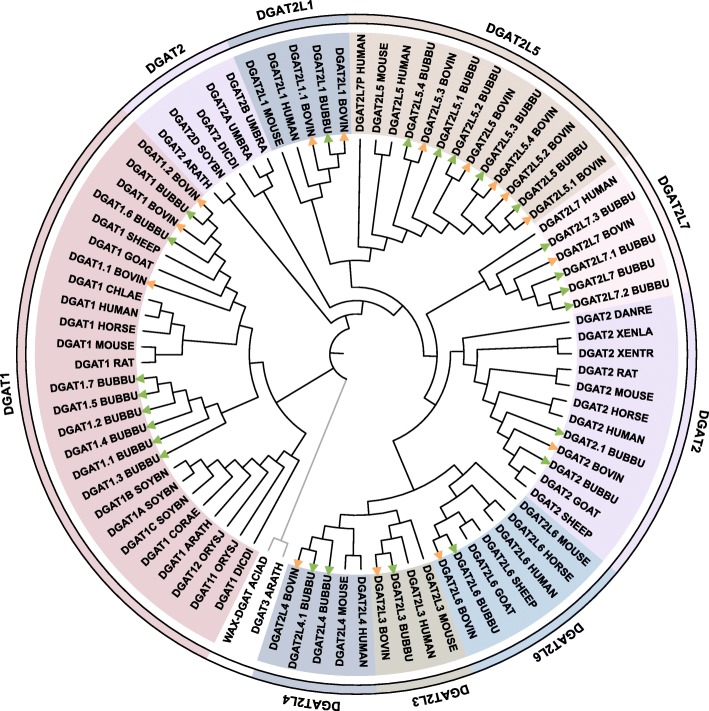
Phylogenetic Neighbor-Joining (NJ) tree of DGAT proteins from different organisms. Identified DGATs in *Bubalus bubalis* (BUBBU) and *Bos taurus* (BOVIN) together with verified DGATs from *Capra hircus* (GOAT), *Ovis aries* (SHEEP), *Equus caballus* (HORSE), *Homo sapiens* (HUMAN), *Mus musculus* (MOUSE), *Rattus norvegicus* (RAT), *Arabidopsis thaliana* (ARATH), *Chlorocebus aethiops* (CHLAE), *Dictyostelium discoideum* (DICDI), *Corylus americana* (CORAE), *Oryza sativa subsp. japonica* (ORYSJ), *Glycine max* (SOYBN), *Danio rerio* (DANRE), *Xenopus laevis* (XENLA), *Xenopus tropicalis* (XENTR), *Umbelopsis ramanniana* (UMBRA) and *Acinetobacter baylyi* (ACIAD) were included in the analyses. The DGAT enzymes are grouped into eight clusters including DGAT1, DGAT2 and DGAT2L1, DGAT2L3, DGAT2L4, DGAT2L5, DGAT2L6 and DGAT2L7, which are represented by different colors. The green and orange arrows represent the identified DGAT proteins isoforms from buffalo and cattle

To assess evolutionary relationships of DGAT proteins in buffalo and other organisms, we conducted a broad phylogenetic analysis of animals (*Bubalus bubalis, Bos taurus, Homo sapiens, Mus musculus, Rattus norvegicus, Capra hircus, Ovis aries, Equus caballus, Chlorocebus aethiops, Danio rerio, Xenopus laevis, Xenopus tropicalis*), plants (*Arabidopsis thaliana, Oryza sativa subsp. japonica, Glycine max, Corylus americana*) and microbes (*Dictyostelium discoideum, Umbelopsis ramanniana, Acinetobacter baylyi*). Accordingly, 85 amino acid sequences containing DGAT proteins from different organisms and all protein isoforms identified in buffalo and cattle, were aligned to generate nonrooted Neighbor-Joining (NJ) tree (Fig. 2) and confirmed by Maximum Likelihood (ML) tree (Additional file 6). Both phylogenetic analyses revealed similar topologies and evolutionary partitioning of DGAT family proteins into two major clades: DGAT1 and DGAT2. While the DGAT3 in *Arabidopsis thaliana* and WAX-DGAT in *Acinetobacter baylyi* constructed an independent branch.

As shown, DGAT1 clade covered DGAT1 in different organisms, but DGAT2 clade consisted of several clusters, including DGAT2, DGAT2L1, DGAT2L3, DGAT2L4, DGAT2L5, DGAT2L6, and DGAT2L7. Moreover, DGAT1 proteins in animals (buffalo, cattle, goat, sheep, horse, mouse, rat, monkey, and human) clustered separately from that of plants (*Arabidopsis*, rice, soya bean, and *Corylus*). Similarly, DGAT2 proteins from animals, plants, and microbes bunched, respectively. Except for DGAT2, other seven DGAT2 family members only existed vertebrate taxa, rather than in an invertebrate. Comparing each member either in DGAT1 or DGAT2 subfamily, the evolutionary relationship between buffalo and cattle was particular closer than buffalo with any other organisms.

### Chromosomal distribution and collinearity analysis of DGAT genes

**Fig. 3:**
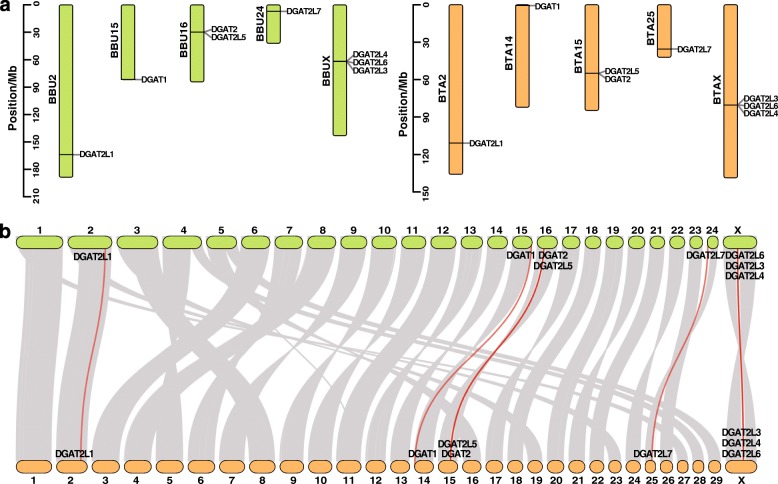
Chromosomal distribution and collinearity analysis of DGAT genes in buffalo and cattle. **a**. Position of DGATs map on buffalo chromosome (BBU) and cattle chromosome (BTA). **b**. The collinear block linkages between *Bubalus bubalis* and *Bos taurus* genome. The ellipse coloured with green and orange represent BBU and BTA, respectively. Two genes link to each other by the grey lines are syntenic genes and the red lines represent syntenic DGATs

Based on genes mapping information of buffalo chromosome (BBU) and cattle chromosome (BTA), eight buffalo DGAT genes distribute on five chromosomes including BBU2 (1), BBU15 (1), BBU16 (2), BBU24 (1) and BBUX (3), and cattle DGATs located on BTA2 (1), BTA14 (1), BTA15 (2), BTA25 (1) and BTAX (3) (Fig. 3a). Among them, buffalo *DGAT2L1* has a similar position with cattle *DGAT2L1* on the second chromosome at 163.89–163.93 Mb and 110.84–110.88 Mb. Whereas the location of *DGAT1* was different between two species, at the end region (81.68–81.69 Mb) of BBU15 in buffalo, but at the start region (0.60–0.61 Mb) of BTA14 in cattle. *DGAT2* and *DGAT2L5* were tandem genes in buffalo at the location of 29.66–29.69 Mb and 29.72–29.92 Mb on BBU16. In contrast, positions of these two genes exchanged in cattle on BTA15, that *DGAT2L5* and *DGAT2* located at 54.98–55.12 Mb and 55.16–55.19 Mb. What is more, DGAT2L3, DGAT2L4, and DGAT2L6 were three X-chromosome linear genes, which located within 80.20–80.40 Mb region for buffalo, and at the region of 61.78–61.98 Mb for cattle.

Collinearity analysis of the genome between buffalo and cattle resulted in the identification of 45,021 pairs of collinear genes (Fig. 3b). The syntenic genes blocks almost covered all the chromosomes between buffalo and cattle. Although the number of chromosomes is different between river buffalo (2 *N* = 50) and cattle (2 *N* = 60), a large chromosome homologous existed between two species. BBU1 appears to be a fusion of BTA1 and BTA27, BBU2 equals to BTA2 and BTA 23, BBU3 equals BTA8 and BTA 19, BBU4 equals BTA5 and BTA 28, and BBU5 equals BTA16 and BTA 29 references to the state of collinear banding. Furthermore, the chromosomes where DGAT genes located in, BBU2, BBU15, BBU16, BBU24, and BBUX have one to one match to BBU2, BTA14, BTA15, BTA25, and BTAX, respectively. Among the DGAT family genes, *DGAT2L1* was collinear between buffalo and cattle, the other seven pairs of DGAT genes were syntenic with each other.

### Haplotype association analyses for buffalo milk production traits

**Fig. 4:**
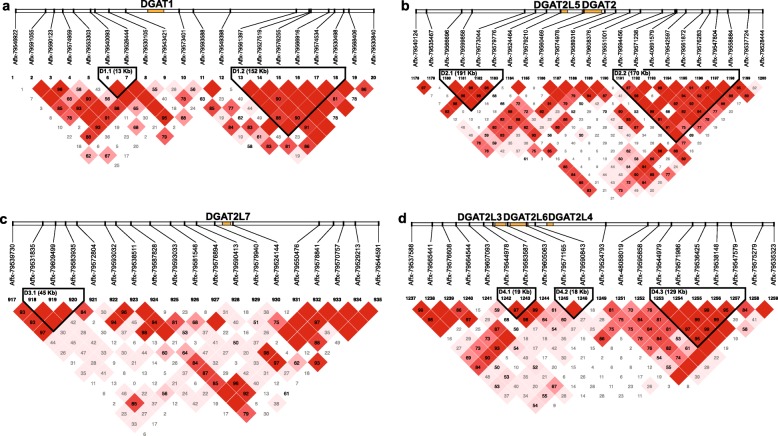
Haplotype blocks pattern in buffalo DGAT located genomic regions based on linkage disequilibrium (LD). **a.** DGAT1 genomic region. **b.** DGAT2 (or DGAT2L5) genomic region. **c.** DGAT2L7 genomic region. **d.** DGAT2Ln (or DGAT2L3, DGAT2L6 and DGAT2L4) genomic region. The numbers on the top indicate the SNP order on the chromosome; SNPs grouped in each triangle box mean they are grouped in one block based on LD (squared correlation coefficient, r2)

To determine whether there are any DGAT family genes are genetically associated with milk production traits in buffalo, we employed the genotypic and phenotypic datasets of 489 buffalos with 1424 lactation records reported in our previous study [4]. Based on the genotyping data after quality control, single nucleotide polymorphisms (SNPs) in the 0.5 Mb genomic region upstream and downstream of each DGAT were filtered and used in the present study [24]. As the results, there were 20, 23, 19, and 23 SNPs identified within the DGAT1, DGAT2 and DGAT2L5, DGAT2L7, DGAT2L3, DGAT2L6, and DGAT2L4 (DGAT2Ln) genomic windows, respectively. The linkage disequilibrium (LD) relationships among SNPs were determined to construct haplotype blocks (Fig. 4). Therefore, two haplotype blocks (D1.1 and D1.2) recognized in DGAT1 genomic region. The first one spanning 13 Kb consisted of 2 SNPs and the second block with 6 SNPs spanned 152 Kb. In the tandem region of DGAT2 and DGAT2L5, we detected two blocks (D2.1 and D2.2) with a length of 191 Kb (4 SNPs) and 170 Kb (8 SNPs) as well. Within the DGAT2L7 genomic region window, only one haplotype block (D3.1) constructed among three SNPs (45 Kb). For the three X-chromosome linear genes *DGAT2L3*, *DGAT2L6*, and *DGAT2L4*, three haplotype blocks including D4.1, D4.2 and D4.3 built in the combined genomic region. Interestingly, the second block (D4.2) constituent SNP (Affx-79,571,165) embraced inside of *DGAT2L6*.

For each haplotype block detected in the DGATs extended regions, we performed haplotype association analyses with six buffalo milk production traits including 270-day adjusted peak milk yield (PM270), total milk yield (MY270), fat yield (FY270), fat percentage (FP270), protein yield (PY270), and protein percentage (PP270) (Table 2). Thus, the D1.2 haplotype block in *DGAT1* genomic region displayed to have significant effects on the FP270 and PP270, and the least square mean (LSM) of FP270 (8.02 ± 0.1%) and PP270 (4.51 ± 0.03%) for diplotype H2H2 were lowerst among all haplotype combinations. The D2.1 haplotype block next to *DGAT2* and *DGAT2L5* were identified to influence the peak milk yield, total milk yield, protein yield, and protein percentage. For this block, buffalos with H2H3 diplotype showed the lower LSM of milk yield (2705.0 ± 66.4 Kg) and protein yield (128.0 ± 2.9 Kg) but had the higher protein percentage (8.46 ± 0.12%) compared to that of some other diplotypes. For D2.2 block, buffalo individuals had H2H2 diplotypes with the high total milk yield (3080.0 ± 68.3 Kg), fat yield (256.0 ± 6.3 Kg) and protein yield (144.0 ± 3.0 Kg). Within the genomic window of *DGAT2L7*, the D3.1 haplotype was associated with all of the six milk production traits in buffalo. Moreover, the H3H3 haplotype combination could be the favorable diplotype in the dairy buffalo breeding program, which showed the high peak milk yield (16.6 ± 0.64 Kg), total yield (3248.0 ± 124.0 Kg), fat yield (300.0 ± 11.5 Kg) and protein yield (157.0 ± 5.5 Kg) and had the high milk fat percentage (9.25 ± 0.22%) and protein percentage (4.84 ± 0.06%). Three adjacent haplotype blocks on chromosome X were detected to influence different milk production traits: D4.1 mainly had effect on MY270, FY270 and PP270, and its H1H1 diplotype showed high total milk yield (3138.0 ± 146.0 Kg) and fat yield (260.0 ± 13.2 Kg); D4.2 had effect on FY270, FP270, PY270 and PP270, but its H1H1 diplotype showed low fat yield (220.0 ± 8.8 Kg), fat percentage (7.94 ± 0.17%), protein yield (128.0 ± 4.3 Kg), protein percentage (4.58 ± 0.05%); D4.3 only had some effects on PP270.

**Table 2:** Haplotype association analyses for six milk production traits in buffalo

Blocks	Haplotype	Frequency	Haplotype combination (n)	Traits (LSM ± SE)					
				PM270/Kg	MY270/Kg	FY270/Kg	FP270/%	PY270/Kg	PP270/%
D1.1	H1: TA	38.16%	H1H1 (55)	15.4 ± 0.26	3073.0 ± 50.6	258.0 ± 4.6	8.40 ± 0.09	144.0 ± 2.2	4.67 ± 0.03
	H2: TT	31.23%	H1H2 (106)	15.0 ± 0.19	3007.0 ± 37.7	243.0 ± 3.4	8.13 ± 0.07	139.0 ± 1.7	4.64 ± 0.02
	H3: CA	30.61%	H1H3 (100)	15.2 ± 0.21	3073.0 ± 40.8	254.0 ± 3.7	8.31 ± 0.07	141.0 ± 1.8	4.60 ± 0.02
			H2H2 (35)	15.0 ± 0.34	2993.0 ± 67.0	247.0 ± 6.1	8.26 ± 0.12	140.0 ± 2.9	4.69 ± 0.03
			H2H3 (77)	15.2 ± 0.22	3056.0 ± 43.3	251.0 ± 4.0	8.27 ± 0.08	141.0 ± 1.9	4.63 ± 0.02
			H3H3 (37)	15.1 ± 0.35	3119.0 ± 69.8	263.0 ± 6.4	8.41 ± 0.13	145.0 ± 3.1	4.66 ± 0.04
D1.2	H1: AATGAC	37.30%	H1H1 (56)	14.9 ± 0.23	2976.0 ± 45.3	250.0 ± 4.1	**8.43 ± 0.08**^**a**^	139.0 ± 2.0	**4.69 ± 0.02**^**a**^
	H2: GATGGC	25.73%	H2H1 (75)	15.3 ± 0.19	3031.0 ± 37.5	248.0 ± 3.4	8.19 ± 0.07	141.0 ± 1.6	**4.65 ± 0.02**^**a**^
	H3: GACGAC	21.65%	H2H2 (34)	15.5 ± 0.27	3142.0 ± 53.1	251.0 ± 4.8	**8.02 ± 0.10**^**b**^	141.0 ± 2.3	**4.51 ± 0.03**^**b**^
			H3H1 (67)	14.8 ± 0.21	3013.0 ± 41.3	251.0 ± 3.8	**8.39 ± 0.08**^**a**^	141.0 ± 1.8	**4.68 ± 0.02**^**a**^
			H3H2 (40)	15.3 ± 0.25	3080.0 ± 49.0	255.0 ± 4.5	8.29 ± 0.09	142.0 ± 2.2	4.62 ± 0.03
			H3H3 (16)	15.2 ± 0.43	3079.0 ± 85.3	261.0 ± 7.8	8.47 ± 0.15	146.0 ± 3.7	**4.74 ± 0.04**^**a**^
D2.1	H1: CGTT	44.68%	H1H1 (84)	14.7 ± 0.27	**2935.0 ± 52.3**^**a**^	238.0 ± 4.8	8.15 ± 0.1	136.0 ± 2.3	**8.15 ± 0.10**^**a**^
	H2: TATC	42.60%	H2H1 (152)	**15.0 ± 0.23**^**a**^	**2968.0 ± 44.0**^**a**^	244.0 ± 4.0	8.27 ± 0.08	137.0 ± 1.9	**8.27 ± 0.08**^**ac**^
	H3: TAGC	9.95%	H2H2 (72)	**15.0 ± 0.25**^**a**^	2988.0 ± 48.0	245.0 ± 4.4	8.25 ± 0.09	**139.0 ± 2.1**^**a**^	8.25 ± 0.09
			H2H3 (34)	**13.8 ± 0.34**^**b**^	**2705.0 ± 66.4**^**b**^	228.0 ± 6.1	8.46 ± 0.12	**128.0 ± 2.9**^**b**^	**8.46 ± 0.12**^**b**^
			H3H1 (37)	14.4 ± 0.34	2811.0 ± 66.0	232.0 ± 6.1	8.30 ± 0.12	130.0 ± 2.9	8.30 ± 0.12
			H3H3 (8)	15.0 ± 0.68	2903.0 ± 131.9	240.0 ± 12.1	8.30 ± 0.24	141.0 ± 5.8	**8.30 ± 0.24**^**b**^
D2.2	H1: TATTCCT	69.04%	H1H1 (189)	14.9 ± 0.19	2969.0 ± 36.3	243.0 ± 3.3	8.19 ± 0.07	138.0 ± 1.6	8.19 ± 0.07
	H2: GGCCGTG	21.20%	H1H2 (117)	14.6 ± 0.17	2911.0 ± 33.5	243.0 ± 3.1	8.37 ± 0.06	137.0 ± 1.5	8.37 ± 0.06
	H3: TATTGTT	6.71%	H1H3 (44)	14.4 ± 0.30	2819.0 ± 58.9	**231.0 ± 5.4**^**a**^	8.23 ± 0.11	133.0 ± 2.6	8.23 ± 0.11
			H2H2 (23)	15.1 ± 0.35	**3080.0 ± 68.3**^**a**^	**256.0 ± 6.3**^**b**^	8.33 ± 0.12	**144.0 ± 3.0**^**a**^	8.33 ± 0.12
			H3H2 (10)	13.9 ± 0.59	**2674.0 ± 114.3**^**b**^	227.0 ± 10.4	8.51 ± 0.21	**126.0 ± 5.0**^**b**^	8.51 ± 0.21
			H3H3 (3)	14.9 ± 0.90	2855.0 ± 175.4	228.0 ± 16.0	8.10 ± 0.32	134.0 ± 7.7	8.10 ± 0.32
D3.1	H1: ACG	51.88%	H1H1 (116)	**15.1 ± 0.14**^**a**^	2973.0 ± 27.1	**248.0 ± 2.5**^**a**^	**8.34 ± 0.05**^**a**^	**139.0 ± 1.2**^**a**^	**4.67 ± 0.01**^**a**^
	H2: GTA	24.53%	H1H2 (92)	**14.3 ± 0.17**^**b**^	**2864.0 ± 31.9**^**a**^	**236.0 ± 3.0**^**b**^	**8.27 ± 0.06**^**a**^	**134.0 ± 1.4**^**ac**^	**4.69 ± 0.02**^**a**^
	H3: GCG	11.88%	H1H3 (42)	**15.3 ± 0.23**^**a**^	**3071.0 ± 43.8**^**b**^	**253.0 ± 4.1**^**a**^	**8.28 ± 0.08**^**a**^	**141.0 ± 2.0**^**ab**^	**4.63 ± 0.02**^**ab**^
			H2H2 (24)	15.1 ± 0.30	**3075.0 ± 58.6**^**b**^	**250.0 ± 5.4**^**ab**^	**8.15 ± 0.11**^**a**^	141.0 ± 2.6	4.61 ± 0.03
			H2H3 (25)	15.2 ± 0.30	**3090.0 ± 57.5**^**b**^	**260.0 ± 5.3**^**a**^	**8.41 ± 0.10**^**a**^	140.0 ± 2.6	**4.56 ± 0.03**^**b**^
			H3H3 (6)	**16.6 ± 0.64**^**b**^	**3248.0 ± 124.0**^**b**^	**300.0 ± 11.5**^**c**^	**9.25 ± 0.22**^**b**^	**157.0 ± 5.5**^**b**^	**4.84 ± 0.06**^**c**^
D4.1	H1: AG	77.51%	H1H1 (243)	15.3 ± 0.76	**3138.0 ± 146.0**^**a**^	**260.0 ± 13.2**^**a**^	8.3 ± 0.26	142.0 ± 6.5	**4.53 ± 0.07**^**a**^
	H2:GT	13.96%	H1H2 (89)	15.3 ± 0.76	**2988.0 ± 148.0**^**b**^	**248.0 ± 13.4**^**b**^	8.31 ± 0.26	138.0 ± 6.5	**4.64 ± 0.07**^**b**^
	H3:GG	8.06%	H1H3 (51)	14.8 ± 0.78	2985.0 ± 152.0	**243.0 ± 13.7**^**b**^	8.2 ± 0.27	134.0 ± 6.7	**4.51 ± 0.08**^**ac**^
			H2H2 (10)	14.7 ± 0.99	3020.0 ± 191.0	246.0 ± 17.3	8.19 ± 0.34	144.0 ± 8.5	**4.74 ± 0.10**^**b**^
			H3H2 (11)	15.2 ± 0.97	2991.0 ± 187.0	242.0 ± 17.1	8.1 ± 0.34	138.0 ± 8.3	4.62 ± 0.09
			H3H3 (2)	12.5 ± 2.07	2360.0 ± 400.0	180.0 ± 36.3	7.74 ± 0.71	103.0 ± 17.7	4.40 ± 0.20
D4.2	H1:TC	74.50%	H1H1 (240)	14.1 ± 0.50	2797.0 ± 96.7	**220.0 ± 8.8**^**a**^	**7.94 ± 0.17**^**a**^	**128.0 ± 4.3**^**a**^	**4.58 ± 0.05**^**a**^
	H2: CT	25.24%	H1H2 (135)	14.2 ± 0.50	2851.0 ± 96.2	**235.0 ± 8.7**^**b**^	**8.25 ± 0.17**^**b**^	**133.0 ± 4.3**^**b**^	**4.64 ± 0.05**^**b**^
			H2H2 (31)	14.9 ± 0.54	2901.0 ± 105	**239.0 ± 9.5**^**b**^	8.23 ± 0.19	**136.0 ± 4.6**^**b**^	**4.68 ± 0.05**^**b**^
D4.3	H1: CGGTC	51.44%	H1H1 (108)	15.0 ± 0.77	2918.0 ± 148.0	242.0 ± 13.5	8.3 ± 0.27	135.0 ± 6.6	**4.64 ± 0.07**^**a**^
	H2: TATCT	37.75%	H1H2 (153)	14.7 ± 0.76	2885.0 ± 148.0	235.0 ± 13.4	8.16 ± 0.26	134.0 ± 6.5	**4.65 ± 0.07**^**a**^
	H3: CGGCT	8.43%	H1H3 (39)	14.8 ± 0.80	2899.0 ± 155.0	239.0 ± 14.1	8.25 ± 0.28	132.0 ± 6.9	**4.54 ± 0.08**^**b**^
			H2H2 (63)	14.7 ± 0.79	2886.0 ± 152.0	231.0 ± 13.8	8.04 ± 0.27	133.0 ± 6.7	4.62 ± 0.08
			H3H2 (25)	14.6 ± 0.82	2946.0 ± 159.0	236.0 ± 14.4	8.06 ± 0.28	134.0 ± 7.0	4.56 ± 0.08
			H3H3 (2)	13.9 ± 2.05	2949.0 ± 397.0	237.0 ± 35.9	8.02 ± 0.71	131.0 ± 17.5	4.42 ± 0.20

Frequency (%), the frequency of individuals with each haplotype among population; Haplotype combination (n), the number of individuals with each haplotype combination among population; LSM ± SE represent the least square means ± standard error; PM270, 270-day peak milk yield; MY270, 270-day total milk yield; FY270, 270-day fat yield; FP270, 270-day fat percentage; PY270, 270-day protein yield and PP270, 270-day protein percentage. For each haplotype block each trait, values with different superscripts differ significantly at Bonferroni corrected *P* < 0.05

### Candidate makers affecting buffalo milk production traits

**Fig. 5:**
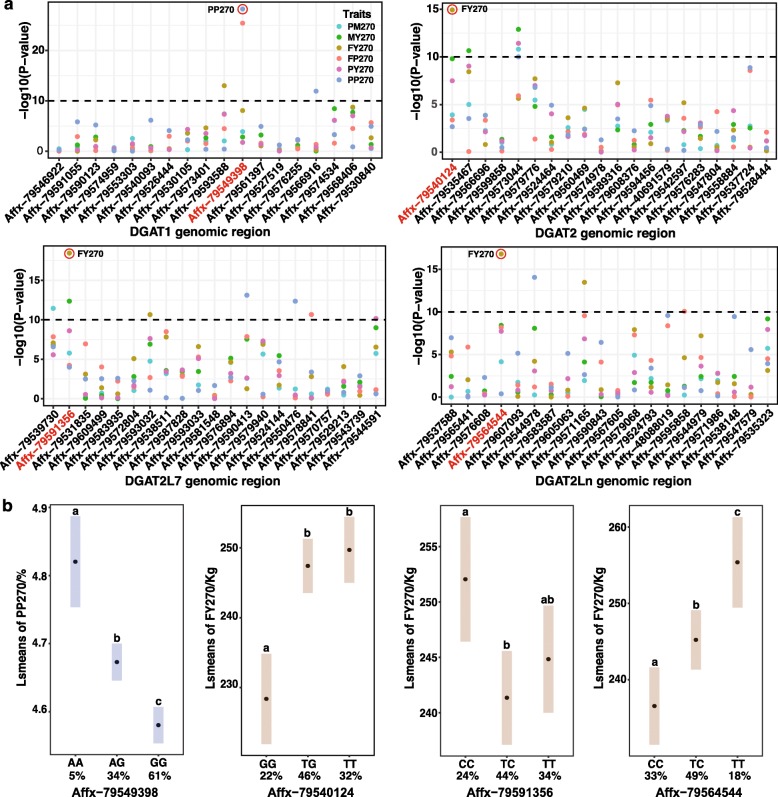
SNP association analyses and most significant SNP influencing milk proudction traits in buffalo. **a.** Single marker association with buffalo milk production traits. PM270, 270-day peak milk yield; MY270, 270-day total milk yield; FY270, 270-day fat yield; FP270, 270-day fat percentage; PY270, 270-day protein yield; PP270, 270-day protein percentage. **b.** Least square mean (LSM) of the three genotypes affecting PP270 or FY270. Different letters above represent significant (Bonferroni corrected *P* < 0.05) difference between two genotypes, and same letters mean insignificant difference. Number under each genotype represent the percentage of buffalo individuals with that genotype to total number of buffalos

With the single marker and single trait association between SNPs and buffalo milk production traits, we identified the most significant SNP in each objective region (0.5 Mb upstream and downstream of each DGATs) (Fig. 5). Accordingly, a total of 20 SNPs was identified to be associated with different milk production traits at the level of *P*-value < 10^− 10^. As the most significant SNP in the DGAT1 genomic region, Affx-79,549,398 presented to have impacts on buffalo milk protein percentage and fat percentage. The other three SNPs, Affx-79,540,124 (DGAT2 or DGAT2L5 genomic region), Affx-79,591,356 (DGAT2L7 genomic region) and Affx-79,564,544 (DGAT2Ln genomic region), all were associated with milk fat yield of buffalo.

For each of the four SNPs, we further calculated the least square mean of the three genotypes affecting the trait, in order to investigate their genetic contribution. At the locus of Affx-79,549,398 affecting PP270, buffalo individuals with AA (4.90 ± 0.05%) genotype have the significant higher protein percentage than that of AG (4.76 ± 0.04%) and GG (4.67 ± 0.04%) genotypes. Hence, buffalo with AA genotype can be selected to improve the protein percentage in milk effectively. In the studied buffalo population, individuals with AA genotyped only occupied 5% of the total population. The low frequency of the favorable genotype provides more opportunity for improvement in the whole buffalo population. For the Affx-79,540,124, the 270-day milk fat yield produced by buffalos with TT (250 ± 2.41 Kg) and TG (228 ± 3.30 Kg) were both significantly higher than that of GG genotype (247 ± 1.98 Kg). At the Affx-79,591,356 locus, individuals with CC genotype (252 ± 2.87 Kg) had a significant higher fat yield in buffalo milk compared with that of TC genotypes (241 ± 2.16 Kg). Conclusively, AX-85063131 affecting FY270 showed that the individuals with TT homozygous genotype showed the highest milk fat yield (255 ± 3.02 Kg) among all three genotypes. These results indicated that G allele at Affx-79540124 locus and T allele at Affx-79564544 seem to be the favorable alleles used to improve the milk fat yield in the buffalo breeding program.

## Discussion

Water buffalo is an important livestock species for the agricultural economy, supplying milk, meat, and draught power [25]. As known, cattle and buffalo ranked as the first and second milk source worldwide. *DGAT1* and *DGAT2* are essential genes related to milk traits in cattle and buffalo. The DGAT family genes encoding functional proteins have been well characterized in many plants recently [13, 23]. However, investigation of DGAT gene family in animals, especially in buffalo was limited. Since buffalo and cattle are closely related, the vast amount of cattle genetic resources might serve as references for the buffalo community to further advance genome science in the species [26].

### Structural features of buffalo DGAT family proteins and genes

In this study, we performed a genome-wide detection and identification of eight DGAT family genes from the first complete genome of buffalo (UOA_WB_1 assembly) [22] and the latest genome version of cattle (ARS-UCD1.2 assembly). Among eight buffalo DGATs, *DGAT1* belongs to the DGAT1 subfamily, and the DGAT2 subfamily consisted of *DGAT2, MOGAT1, MOGAT2, MOGAT3, AWAT1, AWAT2,* and *DGAT2L6*. Each of the DGATs was found to encode several protein sequences owing to undergoing alternative splicing and intron retention. It may be a regulatory pathway to control the amount of active DGAT enzymes, providing a possible molecular mechanism for increased triacylglycerol biosynthesis [27].

DGAT enzymes activities are mostly dependent on their functional motifs and domains in the proteins. The distribution of the conserved motifs of DGAT1 subfamily proteins was different from that in DGAT2 subfamily members, meaning some specific functions may exist between the two subfamilies. Interestingly, four conserved amino acid sequences in Motif 1, Motif 3, Motif 5, and Motif 9 were well-kept among all buffalo DGAT family members. This result was consistent with the previous study [28] that three conserved motifs have been identified in DGAT family proteins from different organisms, including plants, animals, fungi, and human. These highly conserved motifs may be located at the active sites of the enzymes and play essential roles in structure, substrate binding, and catalysis. Several protein isoforms like DGAT1.5 and DGAT1.7, lack of conserved amino acid residues indicated some sequence loss occurred in the evolution of DGAT1 ancestor gene.

Furthermore, the gene structural analyses showed that the number and distribution of CDSs, introns, and UTRs were divergent among buffalo DGAT1 and DGAT2 subfamily genes. Inside of each subfamily, the important coding sequences of genes that translated proteins were similar. While the vast dissimilarities in the length and layout of introns and UTRs, which should be the main reason contributing to gene structure variances. Overall, buffalo DGAT1 subfamily member does not share extensive conversed nucleic acid sequence and amino acid similarities with DGAT2 subfamily members. This finding has suggested that have different functions and could play non-redundant roles in buffalo.

### Phylogenetic relationship of DGAT family proteins

The phylogenetic analysis of DGAT family proteins in different organisms provided an in-deep insight on their evolutionary relationships among species. Consistent with the initial classification, DGAT1, and DGAT2 subfamily proteins were clustered in two different clades, indicating that they have been evolved asymmetrically. Besides the two main clades, DGAT3 and WAX-DGAT, formed a monophyletic subgroup, which has not detected in buffalo and cattle. Refer to the relevant studies, and we discovered that DGAT3 and WAX-DGAT are two types of DGAT enzymes that phylogenetically divergent from DGAT1 and DGAT2 [13, 23]. These two genes mostly identified in plants and yeast, but rarely found in animals [16, 28]. On the contrary, DGAT1 and DGAT2 almost ubiquitously found in animals and plants and was readily identifiable in the major eukaryote [16]. However, the DGAT1 or DGAT2 protein from animals and plants clustered, respectively. The phenomenon reflected the evolutionary pattern and classification of DGAT protein family were actual differences between animals and plants. Among all organisms, DGAT family proteins in buffalo and cattle showed high sequence similarity and close phylogenetic relationship, strongly suggested that the DGAT family genes between two species have similar functions.

### Collinearity analysis of DGATs in buffalo and cattle

Chromosomal distributions of the DGAT family genes showed that eight genes located on five different chromosomes both in buffalo and cattle. The family genes distributed on different chromosomes generally designated as segmental duplication events, and those co-located on the same chromosome are considered tandem duplication events [29]. Hence, both tandem and segmental duplication events have happened for the expansion of DGAT genes in the genome of buffalo and cattle. While the whole cattle genome consists of 29 autosomes and a pair of X/Y, of sex chromosomes, the river buffalo has 24 autosomes plus the X and Y chromosomes. From the collinearity analysis results, we can find large homologous chromosomal regions that existed between the two species. The cytogenetic studies [30] and genetic mapping [31] have demonstrated the same results that chromosomes can be matched arm for arm between buffalo and cattle. The chromosomes were homologous in the two species, but the positions where DGAT genes located in were inconsistent or even opposite, such as *DGAT1* and *DGAT2L7*. Each pair of DGAT genes between buffalo and cattle either were syntenic (not necessarily in the same order) or collinear (conserved in the same order). Some discrepancies in the order of the gene might occur because of chromosome rearrangement in many years of evolution. In total, the extensive homology between buffalo and cattle provided rich perspectives for studying DGAT family genes function in buffalo.

### Candidate DGAT genes affecting milk production traits in buffalo

To dissect whether any buffalo DGAT genes have effects on the milk production traits, we performed both haplotype association analysis and SNP association analysis to identify the candidate genomic regions, makers, and genes. As the results, seven haplotype blocks and four SNPs were suggested as most possibility markers for influencing buffalo milk performance. As known, the *DGAT1* gene became a robust functional candidate gene for milk fat percentage after the description of the lactation deficiency in DGAT-deficient mice [17]. Two SNPs in the *DGAT1* that cause a missense mutation (K232A) showed to have significantly affect milk fat content and milk yield in cattle [7, 8]. Coherent with the previous study, SNPs nearby *DGAT1* constructed two haplotype blocks presenting to be associated with milk fat percentage in buffalo. The most significant SNP Affx-79,549,398 have impacts on buffalo milk protein percentage and fat percentage. In Mehsana buffalo, one SNP (g.8259G > A) in *DGAT1* was detected to associate with first lactation yield [32]. Unfortunately, due to the minor SNP density of our used genotyping arrays, we have not detected relevant genetic variations within the buffalo *DGAT1* gene. On the other hand, it probably because that polymorphism of K232A in *DGAT1* was fixed on K allele after years of natural selection and artificial selection in buffalo [11, 33], or became a rare sight in buffalo population and filtered in quality control analysis. Analysis in Anatolian buffalo provided evidence that fixed allele concerning *DGAT1* was responsible for the high milk fat yield [12].

Several studies have explored the role of *DGAT2* gene play in affecting lactation performances in buffalo [18], cattle [20], and goat [19]. *DGAT2L5*, also known as *MOGAT2*, which investigated for polymorphisms that might be associated with breeding values for milk fat percentage in some cattle breeds [21]. Some mutations also revealed in buffalo *MOGAT2*. However, there were not significantly different among different genotypes for milk production traits in Murrah buffalo and Indian buffalo population [18, 34]. In our Italian Mediterranean buffalo population, two haplotype blocks next to *DGAT2* and *DGAT2L5* genes were identified to influence most of the six buffalo milk production traits. Although we have not discovered SNP groups with linkage disequilibrium within the genomic window of *DGAT2L1/MOGAT1*, this gene selected as a new promising gene associated with milk fatty acid traits in Chinese Holstein [35, 36]. For *DGAT2L7/MOGAT3, DGAT2L3/AWAT1*, *DGAT2L4/AWAT2* and *DGAT2L6* genes, researches of studying their effects on lactation performance in animals were very limited. *MOGAT3* gene only was reported to have effects on growth traits in Nanyang cattle [37]. Accordingly, *DGAT1*, *DGAT2*, *MOGAT1*, and *MOGAT2* were four essential candidate genes affecting milk production traits both in buffalo and cattle. While four DGAT2 subfamily genes, including *MOGAT3*, *AWAT1*, *AWAT2*, and *DGAT2L6* could be novel candidates, and further investigation and validation are necessary.

The DGAT family genes and the upstream regulated miRNAs together with some interacted genes constructed an integrative network (Additional file 7), that participated in the molecular regulation of triacylglycerol biosynthesis in lipid metabolism process. The functional analysis revealed that DGAT family genes encoding the enzymes directly or indirectly interact with each other, performing non-redundant functions, collectively regulating lipid metabolism, and affecting milk secretion and synthesis in mammals.

## Conclusions

This study performed a comprehensive genome-wide analysis of the DGAT-enzyme genes in buffalo. A total of eight DGAT genes were identified in buffalo and grouped into two distinct clades. Collinearity analysis revealed that DGAT family genes were homologous between buffalo and cattle. Our association analysis and functional prediction indicated that DGAT family genes could be the candidate genes affecting milk production traits in buffalo. Our findings provided an essential lead for further studies of DGAT genes in animals.

## Methods

### Genome-wide identification of DGAT genes

Data resources of the genome, proteome and annotation of buffalo (*Bubalus bubalis*, UOA_WB_1 assembly) and cattle (*Bos taurus*, ARS-UCD1.2 assembly) are from NCBI database (https://www.ncbi.nlm.nih.gov/). In order to identify all the possible DGATs in buffalo and cattle, both the Basic Local Alignment Search Tool (BLAST) and Hidden Markov Model (HMM) searched were performed [38]. The number of 21 reviewed DGATs sequences of bovine (*Bos taurus*), human (*Homo sapiens*), mouse (*Mus musculus*), and rat (*Rattus norvegicus*) were obtained from UniProt database (https://www.uniprot.org/) (Additional file 1). These protein sequences were taken as the query to search for potential candidates via BLASTP with a threshold of e-value = 10^− 6^. Besides, the known DGATs amino acid sequences were aligned and constructed Hidden Markov Model profiles for DGAT homolog sequences detection in HMMER 3.2 (http://hmmer.org/) with the default setting. The candidate sequences obtained from both approaches were considered as identified DGAT homolog sequences. Subsequently, the non-redundant DGAT homologs were subjected to NCBI CD-search [39] and Pfam [40] to confirm the presence of the conserved protein domain. The identified DGAT gene and protein sequences were named a reference to their corresponding marched sequence of human or mouse or rat, and further confirmed by the transcripts and protein productions. The molecular weight and isoelectric point of buffalo DGAT proteins calculated by ExPASy (https://web.expasy.org/protparam/). Subcellular localization predicted by LocTree3 (https://rostlab.org/services/loctree3).

### Phylogenetic analysis

The known DGAT amino acid sequences in different organisms including animals (*Homo sapiens, Mus musculus, Rattus norvegicus, Capra hircus, Ovis aries, Equus caballus, Chlorocebus aethiops, Danio rerio, Xenopus laevis, Xenopus tropicalis*), plants (*Arabidopsis thaliana, Oryza sativa subsp. japonica, Glycine max, Corylus americana*) and microbes (*Dictyostelium discoideum, Umbelopsis ramanniana, Acinetobacter baylyi*) were downloaded from UniProt database (https://www.uniprot.org/) (Additional file 8). The identified amino acid sequences of DGAT in buffalo and cattle together with the known DGATs from other animals, plants and microbes were aligned by ClusalW and constructed a Neighbor-Joining tree in MEGA 7.0 [41]. The bootstrap test implemented with 1000 replication (random seed). In order to verify the reliability of the Neighbor-Joining method, the phylogenetic trees were reconstructed by Maximum Likelihood method in MEGA 7.0 [41].

### Structural features analysis

To further evaluate the structural diversity of buffalo DGAT genes and proteins, a separate phylogenetic Neighbor-Joining tree constructed, and the conserved motifs were detected in MEME 5.0 [42] and visualized in TBtools [43]. The limits on minimum width, maximum width, and the maximum number of motifs were specified as 6, 50, and 10, respectively. Also, coding sequences and corresponding genomic sequences of buffalo DGATs were loaded into the Gene Structure Display Server (GSDS 2.0) [44] to portray the numbers and positions of CDSs and introns graphically. Prediction of transmembrane helixes of buffalo DGAT proteins obtained by using with a representative amino acid sequence of each DGAT genes via the online server PSIPRED 4.0 (http://bioinf.cs.ucl.ac.uk/).

### Chromosomal distribution and collinearity analysis

Positional information of predicted DGAT genes of buffalo and cattle were extracted from the genomic sequence and annotation files and then were visualization in TBtools [43]. The identified buffalo and cattle DGATs were mapping on buffalo and cattle chromosome. Buffalo and cattle genomic comparisons determined by all-against-all BLASTP searches (e-value = 10^− 6^) using the proteome sequences of *Bubalus bubalis* as queries against those of *Bubalus bubalis* and *Bos taurus*. The collinearity analysis between BBUs and BTAs for orthologous genes was conducted using MCScanX toolkit [45]. The results of collinearity analyses and orthologous DGATs between buffalo and cattle were visualized using TBtools [43].

### Haplotype detection within DGAT located regions

Genotypic and phenotypic datasets of 489 Italian Mediterranean buffalos with 1424 lactation records were reported in our previous study [4]. SNPs within 0.5 Mb genomic window around each buffalo DGATs were obtained from the genotyping data (quality controlled) conducting by the 90 K Axiom® Buffalo SNP Array (Additional file 9). Six buffalo milk production traits including peak milk yield, total milk yield, fat yield fat percentage, protein yield, and protein percentage were all adjusted to 270-day record as detailed reported by Liu et al. [4], in order to eliminate effects of environmental factors like lactation herd, year, season, parity and calf gender on milk production traits [46]. Haplotype blocks in the genomic windows were estimation in Haploview 4.2 [47] and PHASE 2.1 [48] with a Bayesian statistical method. For each haplotype block, the frequency of haplotype among buffalo population were calculated and only the major haplotypes (frequency > 5%) were remained for further association study.

### Association analysis for buffalo milk production traits

For each of the genomic windows, the association between single SNP or haplotype with six 270-day adjusted buffalo milk production traits were performed by using least-squares mean algorithm with lsmeans R-package [48]. The SNPs or haplotypes were regarded as fixed factors in the model. Genetic effects of haplotypes on the six milk production traits were presented as LSM ± standard error (SE) [49] and Bonferroni correction for multiple test was applied to the pairwise comparisons among different haplotype combinations for each haplotype block. The significance threshold was set at the corrected *P*-value < 0.05. Furthermore, SNP with the lowest *P*-value in each detected genomic region was selected. At each locus, the frequency of each genotype among buffalo population were calculated. The LSM of affected milk production trait for the three genotypes were compared, and the significance threshold was set at the Bonferroni corrected *P*-value < 0.05. The plots were generated using the ggplot2 package in R software [50]. The genes interaction network was constructed using the Ingenuity Pathways Analysis (IPA) (http://www.ingenuity.com/).

## Supplementary information

**Additional file 1.** 21 DGAT amino acid sequences of cattle, human, mouse and rat obtained from UniProt (https://www.uniprot.org/).

**Additional file 2 **Details of Genome-wide identified DGAT family members in *Bos taurus*.

**Additional file 3.** Conserved domain prediction of buffalo DGAT protein sequences.

**Additional file 4.** Amino acid sequences logos of 10 identified motifs in buffalo DGAT proteins.

**Additional file 5.** Transmembrane structures of buffalo DGAT proteins predicted by PSIPRED (http://bioinf.cs.ucl.ac.uk/).

**Additional file 6.** Phylogenetic maximum likelihood (ML) tree of DGAT proteins from different organisms.

**Additional file 7.** The interaction network for DGAT genes constructed by the Ingenuity Pathway Analysis.

**Additional file 8.** The known DGAT amino acid sequences in different organisms obtained from UniProt (https://www.uniprot.org/).

**Additional file 9.** The identified SNP within 0.5 Mb genomic window around each DGAT in buffalo.

## Data Availability

All data generated or analyzed during this study are included in this published article and its supplementary information files.

## Abbreviations

CDS: Coding sequences
DGAT: Diacylglycerol acyltransferases
FP270: 270-day fat percentage
FY270: 270-day fat yield
HJ: Neighbor-Joining
HMM: Hidden Markov Model
IPA: Ingenuity Pathway Analysis
LD: Linkage disequilibrium
LSM: Least square means
ML: Maximum likelihood
MY270: 270-day total milk yield
PM270: 270-day peak milk yield
PP270: 270-day protein percentage
PY270: 270-day protein yield
SNP: Single nucleotide polymorphisms
UTR: Untranslated region
