# Supplementary figures and images for: Genome-wide identification of Diacylglycerol Acyltransferases (DGAT) family genes influencing Milk production in Buffalo
Source: BMC Genet. 2020 Mar 6;21:26. doi: 10.1186/s12863-020-0832-y (PMC7059399; doi:10.1186/s12863-020-0832-y)

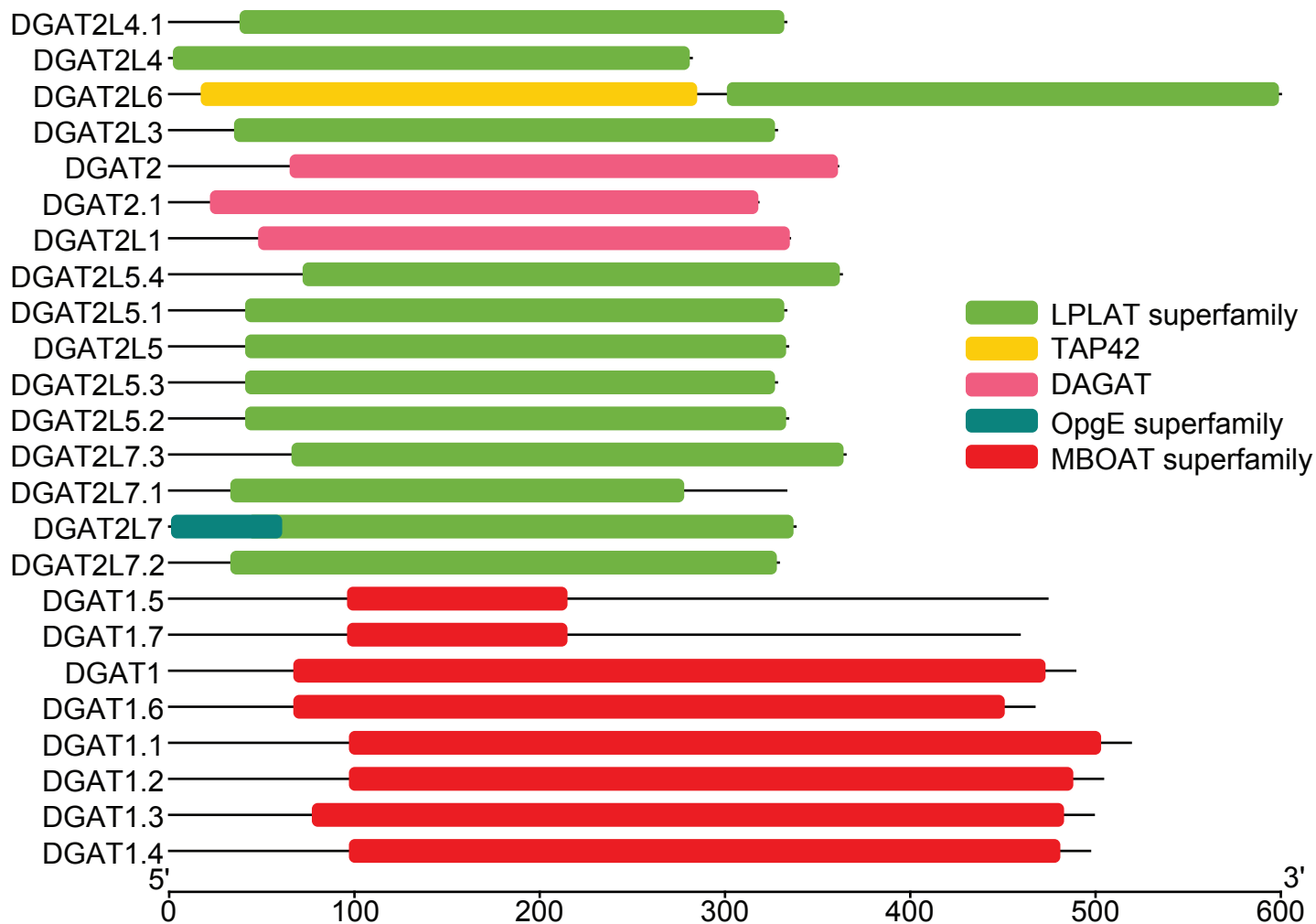

Supplement: Supplementary file 3 — Additional file 3. Conserved domain prediction of buffalo DGAT protein sequences. [file 12863_2020_832_MOESM3_ESM.pdf]

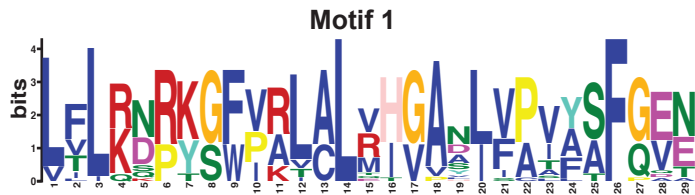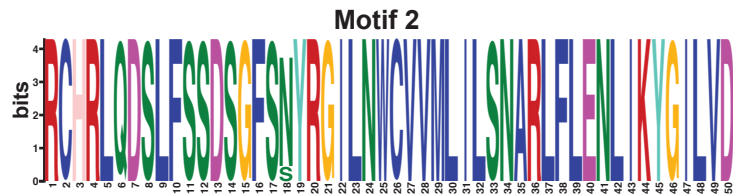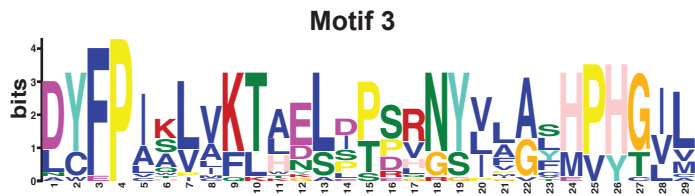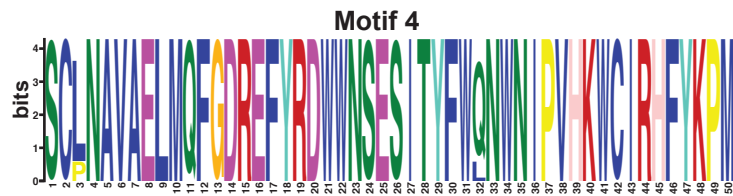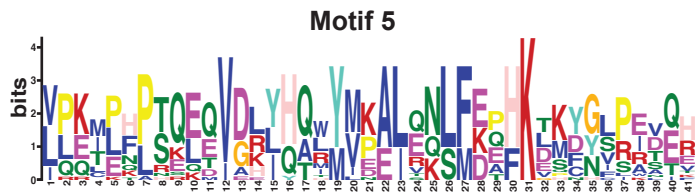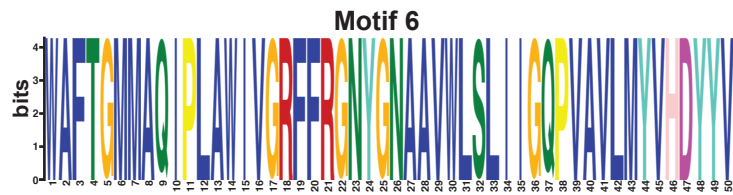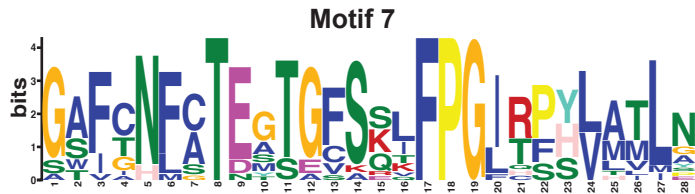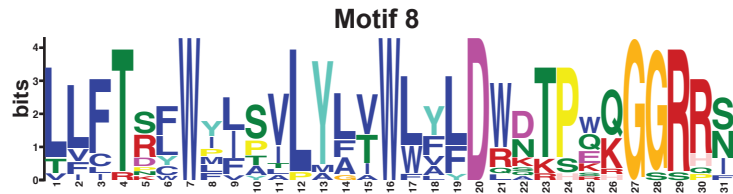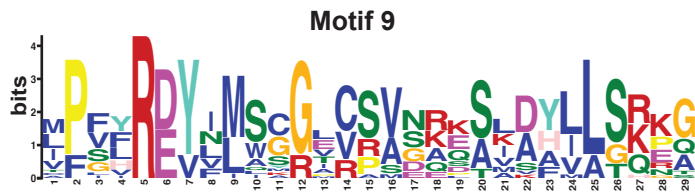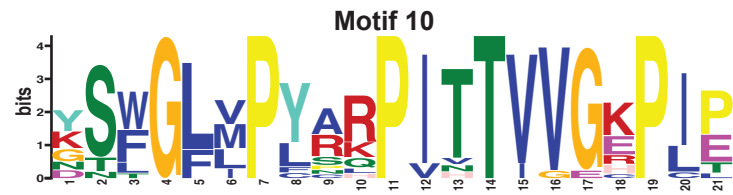

Amino acid sequence

Amino acid sequence

Supplement: Supplementary file 4 — Additional file 4. Amino acid sequences logos of 10 identified motifs in buffalo DGAT proteins. [file 12863_2020_832_MOESM4_ESM.pdf]

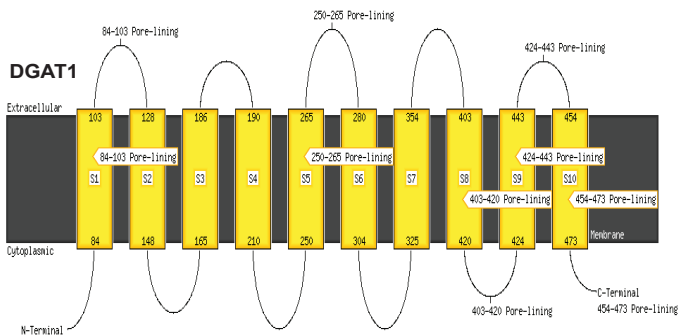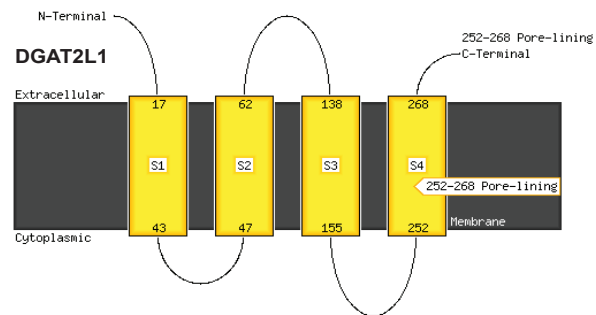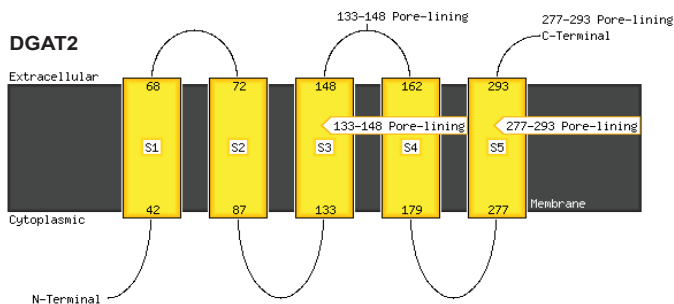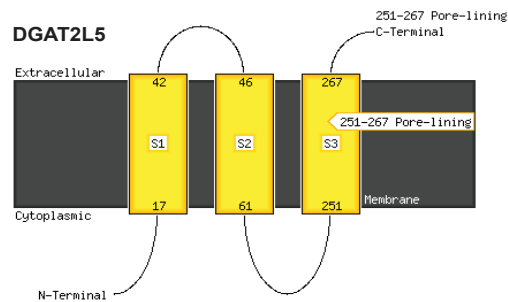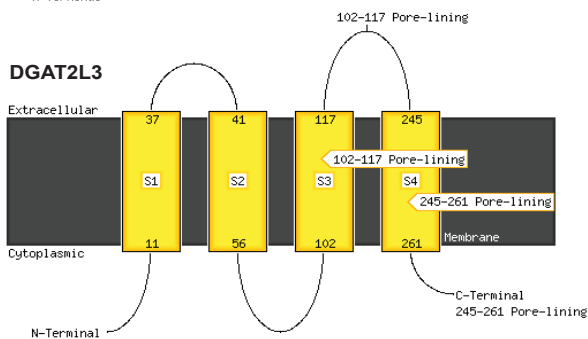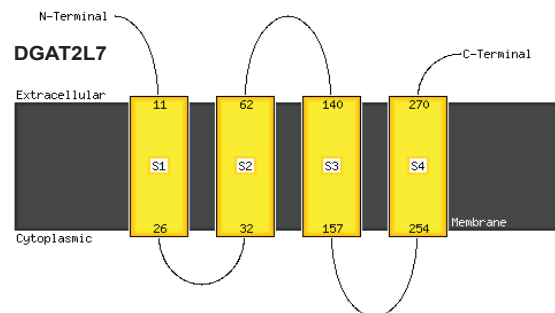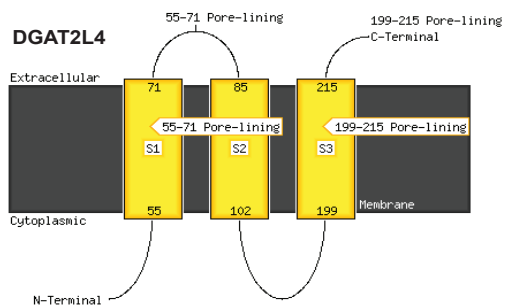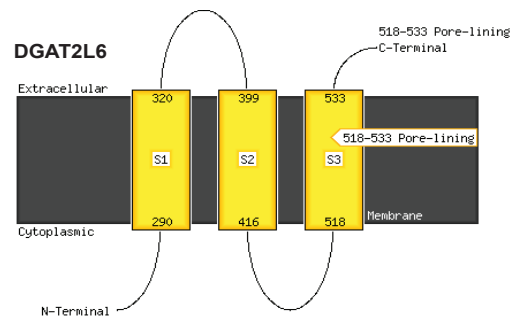

Supplement: Supplementary file 5 — Additional file 5. Transmembrane structures of buffalo DGAT proteins predicted by PSIPRED (http://bioinf.cs.ucl.ac.uk/). [file 12863_2020_832_MOESM5_ESM.pdf]

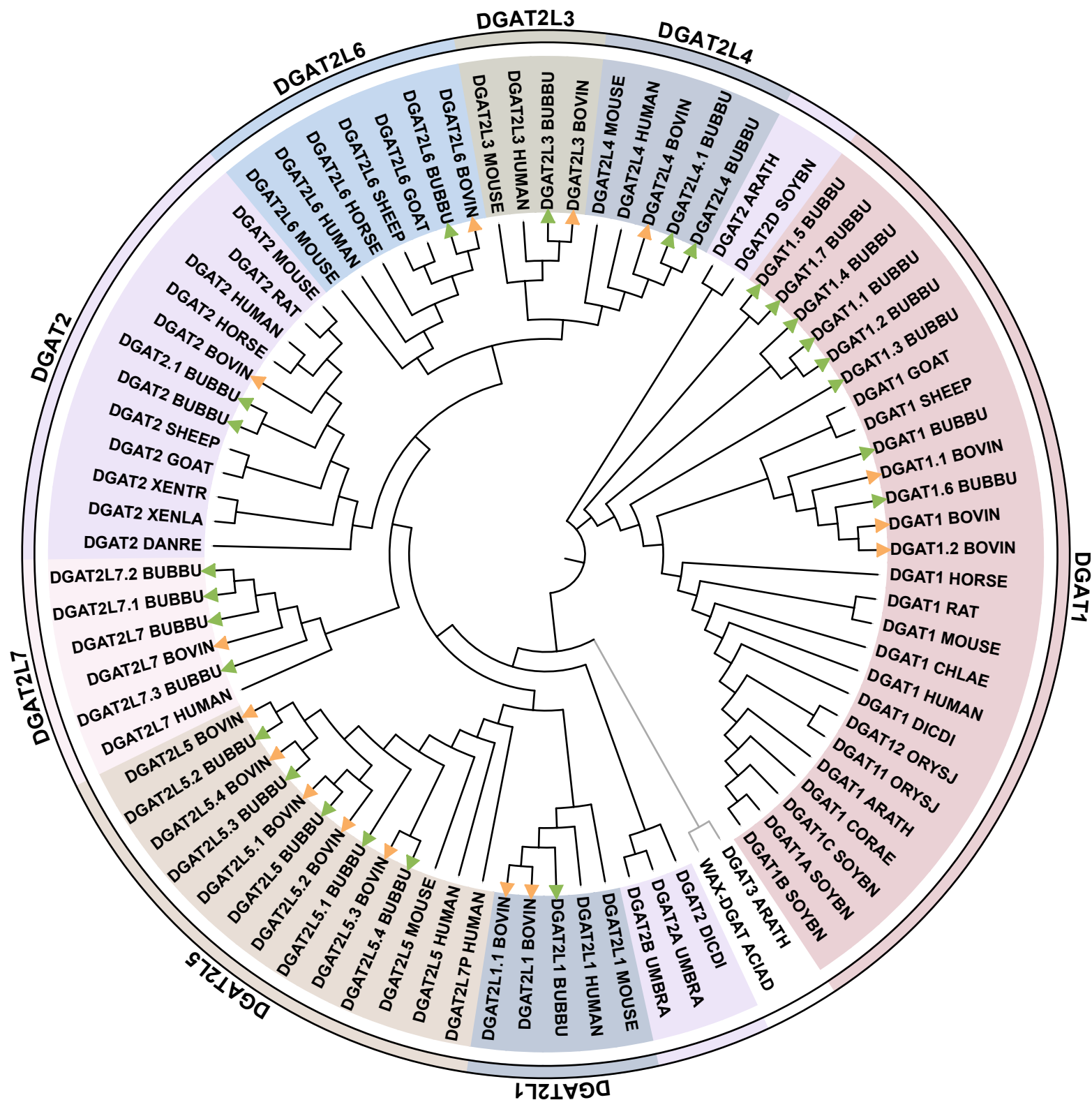

Supplement: Supplementary file 6 — Additional file 6. Phylogenetic maximum likelihood (ML) tree of DGAT proteins from different organisms. [file 12863_2020_832_MOESM6_ESM.pdf]

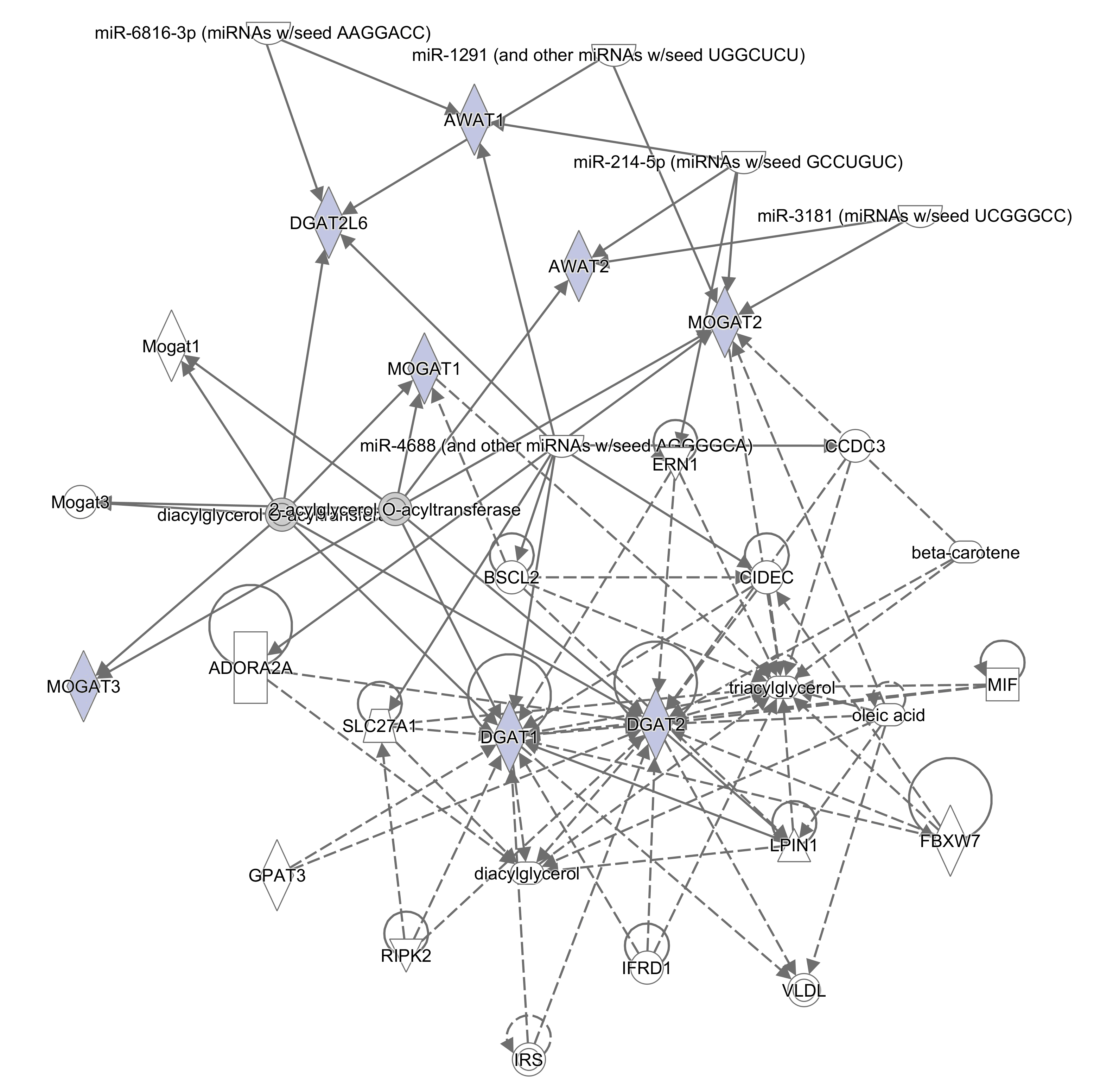

Supplement: Supplementary file 7 — Additional file 7. The interaction network for DGAT genes constructed by the Ingenuity Pathway Analysis. [file 12863_2020_832_MOESM7_ESM.png]
